# Evaluating the Diagnostic Accuracy of Point-of-Care Ultrasound in Acute Abdominal Pain in the Emergency Setting

**DOI:** 10.7759/cureus.92916

**Published:** 2025-09-22

**Authors:** Maham Fatima, Muhammad Farooq, Muhammad Subhan Javed Butt, Muhammad Imran, Wardah Ikram, Aurangzeb Khan, Mohsin Hayat, Atizaz Ali Jan, Ummar Ahmad, Arslan Irshad

**Affiliations:** 1 Department of General Medicine, Gujranwala Medical College, Gujranwala, PAK; 2 Department of Internal Medicine, Nishtar Medical University, Tertiary Care Hospital Nishtar-II, Multan, PAK; 3 Department of Emergency Medicine, Royal Lancaster Infirmary, Lancaster, GBR; 4 Department of Emergency Medicine, Shifa International Hospital, Islamabad, PAK; 5 Department of Internal Medicine, Mohi-ud-Din Islamic Medical College and Teaching Hospital, Mirpur Azad Kashmir, PAK; 6 Department of Acute Medicine, Allama Iqbal Medical College, Lahore, PAK; 7 Department of Surgery, Medical Teaching Institution (MTI) Mardan Medical Complex, Mardan, PAK; 8 Department of Diagnostic Radiology, Tuwaiq Medical Complex, Riyadh, SAU; 9 Department of Trauma and Orthopaedics, University Hospitals Dorset, Poole, GBR; 10 Department of Paediatrics, Osh State Medical University, Osh, KGZ; 11 Department of Paediatrics, Niaz Memorial Hospital, Gujranwala, PAK

**Keywords:** abdominal pain, appendicitis, cholecystitis, emergency department, point-of-care ultrasound, renal colic, sensitivity and specificity

## Abstract

Introduction: Point-of-care ultrasound (POCUS) is increasingly recognized as a valuable diagnostic tool in emergency departments, particularly for evaluating non-traumatic acute abdominal pain. This study assessed the diagnostic accuracy of POCUS in identifying the most common causes of acute abdominal pain, i.e., appendicitis, cholecystitis, bowel obstruction, and renal colic, presenting in an emergency setting.

Methodology: This prospective observational study was conducted over 12 months at Mardan Medical Complex and District Headquarters (DHQ) Teaching Hospital, Gujranwala, Pakistan. A total of 180 adult patients (≥18 years) presenting with non-traumatic acute abdominal pain were enrolled using convenience sampling. POCUS was performed by trained emergency physicians in accordance with American College of Emergency Physicians (ACEP) guidelines. Final diagnoses were confirmed through radiological, surgical, or clinical outcomes. Data were analyzed using SPSS version 26.0 (IBM Corp., Armonk, NY). Statistical measures included sensitivity, specificity, positive predictive value (PPV), negative predictive value (NPV), and diagnostic accuracy.

Results: The mean age of the patients was 39.5 ± 13.6 years (range: 18-80 years), with a slightly higher male predominance: 94 (52.2%) males and 86 (47.8%) females. The most common final diagnoses were appendicitis (44, 24.4%), renal colic (34, 18.9%), cholecystitis (30, 16.7%), and bowel obstruction (28, 15.6%). POCUS correctly identified the pathology in 150 (83.3%) cases. Condition-specific diagnostic accuracies were appendicitis at 92.2%, cholecystitis at 93.7%, bowel obstruction at 94.2%, and renal colic at 93.9%. Cases with nonspecific or unidentifiable abdominal pain were grouped under “Other” and not stratified further. Gender-based accuracy was comparable, with 79 (84.0%) correct diagnoses in males and 71 (82.6%) in females.

Conclusion: POCUS demonstrated high diagnostic accuracy for four of the most common abdominal emergencies, i.e., appendicitis, cholecystitis, bowel obstruction, and renal colic, and showed consistent performance across genders. While these findings support the role of POCUS as a valuable tool in emergency settings, particularly in resource-constrained environments, they should be interpreted within the study’s scope. Broader generalization to all causes of abdominal pain requires further research with larger, more diverse patient populations and long-term follow-up.

## Introduction

Acute abdominal pain is one of the most frequent and challenging presentations encountered in the emergency department (ED), accounting for a significant proportion of emergency visits worldwide [1]. The etiology of abdominal pain can range from benign, self-limiting conditions to life-threatening surgical emergencies, such as appendicitis, perforated viscus, or ruptured abdominal aortic aneurysm [2]. Proper and early diagnosis is important to intervene in time, limit morbidity, and enhance patient outcomes [3]. Nonetheless, the wide-ranging differential diagnosis and clinical manifestations of non-specific nature provide a diagnosis of acute abdominal pain with a dilemma [4]. The current method of diagnosing acute abdominal pain is based on the patient history, physical examination, and a battery of laboratory tests and radiologic imaging that often entail abdominal X-rays, ultrasound, and computed tomography (CT) [5]. Although CT imaging is very specific, it is not always conveniently available, particularly in low-income countries, and exposes patients to radiation and is expensive [6]. The traditional abdominal radiology or conventional abdominal ultrasound that is operated by radiologists has proved to be an effective alternative, although it may be limited by lack of availability, delays, and the need for any operator to be skilled [7].

Point-of-care ultrasound (POCUS) is a type of ultrasound acquired in the bedside setting by the clinician responsible for the care and has represented a fast, low-invasive, less costly, and repeatable imaging method [8]. Over the past years, POCUS has been more commonly used in emergency medicine, where it can be applied in multiple cases, i.e., trauma (Focused Assessment with Sonography in Trauma (FAST) scan), cardiac examination, and assessment of abdominal pathology [9]. It enables emergency doctors to incorporate imaging diagnosis into their clinical experiences in real-time, and this can help in making decisions quickly and in management [10]. The number of studies has shown that POCUS is useful in diagnosing cholecystitis, bowel obstruction, hydronephrosis, and intra-abdominal free fluid [11]. Its current widespread application, notwithstanding its diagnostic accuracy, is still differently reported, and its place vis-à-vis imaging administered by radiologists is still the question [12]. While many international studies have evaluated the performance of POCUS in abdominal emergencies, there is limited local data, especially in resource-constrained healthcare systems like those in Pakistan [13]. Variations in training, equipment, and patient demographics may influence the generalizability of findings from other regions [14]. Moreover, most existing studies focus on specific conditions rather than a comprehensive evaluation of POCUS as a diagnostic tool in undifferentiated acute abdominal pain presentations in the ED setting [15].

The goal of this study was to evaluate the diagnostic accuracy of POCUS in patients who present with acute abdominal pain in an emergency setting because, despite its promising utility, there is a dearth of context-specific evidence regarding the diagnostic accuracy of POCUS in evaluating acute abdominal pain in Pakistani emergency departments.

## Materials and methods

Study design and setting

This prospective observational study was conducted at Mardan Medical Complex and District Headquarters (DHQ) Teaching Hospital, Gujranwala, over a period of 12 months from 22 June 2024 to 22 June 2025.

Sample size calculation

Using a diagnostic test sample size formula, with an expected sensitivity of ~85%, prevalence of ~50%, 95% confidence level, and 7% margin of error [16], a minimum sample size of 165 was calculated. To allow for incomplete data or dropouts, the final sample target was set at 180 patients.

Study population and sampling

Adult patients aged ≥18 years presenting with non-traumatic acute abdominal pain during the study period were consecutively recruited using a convenience sampling approach. A total of 180 participants were enrolled after applying predefined eligibility criteria. Inclusion criteria were age ≥18 years and presentation with non-traumatic acute abdominal pain, while exclusion criteria included pregnancy, hemodynamic instability at presentation, and pre-established diagnoses from other healthcare facilities. All 180 enrolled patients met the inclusion criteria and were included in the final analysis. Convenience sampling was selected as it allowed timely recruitment in a busy emergency department setting and ensured an adequate sample size within the study period.

POCUS protocol, operator training, and data collection

POCUS was performed at the bedside by emergency physicians with at least two years of ultrasound training, following the American College of Emergency Physicians (ACEP) emergency ultrasound guidelines [17]. Operators received formal instruction in visualizing key abdominal structures, including the appendix, focusing on both direct signs (e.g., non-compressible tubular structure >6 mm in diameter) and secondary signs (peri-appendiceal fat changes and free fluid). When direct visualization was not possible, assessment of secondary signs was systematically incorporated to improve diagnostic accuracy. This training was supported and supervised by senior physicians experienced in emergency ultrasonography, including members of the study team such as Dr. Maham Fatima and Dr. Aurangzeb Khan.

A structured protocol evaluated for free intra‑abdominal fluid, biliary tract pathology, hydronephrosis, bowel obstruction, and abdominal aortic aneurysm. A portable ultrasound machine (Mindray M7®, Mindray Bio-Medical Electronics Co., Ltd., Shenzhen, China) with curvilinear and phased‑array transducers was used. The operator recorded POCUS findings immediately and independently, before any radiology or CT imaging results were known.

Patients then underwent standard diagnostic imaging as clinically indicated, including radiology-performed ultrasound and/or contrast‑enhanced CT, along with surgical or follow‑up data as needed. Final diagnoses derived from imaging, operative findings, histopathology, and clinical course served as the reference standard.

Laboratory investigations

Standard blood tests, including complete blood count, liver and renal function tests, and inflammatory markers, were performed as part of routine clinical care based on the physician's discretion. Although these results guided patient management, laboratory data were not systematically collected or analyzed as part of this study’s diagnostic accuracy assessment, which focused primarily on POCUS findings compared to the final clinical diagnosis.

Final diagnosis and follow-up

The final diagnosis for each participant was determined using a composite reference standard that included radiology-performed ultrasound, contrast-enhanced CT, surgical findings, histopathology, and clinical course during the index hospital visit. For admitted patients, the diagnosis was typically confirmed within 24 to 72 hours through imaging and/or operative findings. For those discharged from the emergency department without a definitive diagnosis, a structured seven-day telephone follow-up was conducted to confirm symptom resolution or capture any revised diagnoses based on subsequent healthcare encounters. This systematic approach ensured that all cases, including those with initially non-specific abdominal pain, were accounted for and that no missed diagnoses were overlooked without appropriate follow-up.

Reference standard and quality control

All POCUS scans adhered to standardized practice based on recognized clinical ultrasound parameters (e.g., the American Institute of Ultrasound in Medicine (AIUM) and the American College of Radiology (ACR) practice parameters for diagnostic ultrasound) to ensure consistent technique and interpretation [18]. A senior emergency physician with more than five years of experience randomly reviewed a portion of scans to maintain inter‑observer reliability.

Data analysis

SPSS version 26.0 (IBM Corp., Armonk, NY) was used for the analyses. The data were summarized using descriptive statistics. Categorical factors, such as gender, presenting symptoms, and POCUS diagnostic categories, were expressed as frequencies and percentages, while continuous variables, such as patient age, were given as mean ± standard deviation (SD). Key accuracy parameters, such as sensitivity, specificity, positive predictive value (PPV), negative predictive value (NPV), and overall diagnostic accuracy, were computed by comparing POCUS results to the final validated clinical diagnosis to assess the diagnostic performance of POCUS. Additionally, to evaluate the differential diagnostic efficacy of POCUS across a range of clinical settings, subgroup analyses were conducted for particular abdominal illnesses such as renal colic, cholecystitis, appendicitis, and intestinal obstruction.

Ethical considerations

Ethical approval was obtained from the review boards of both participating institutions: the Institutional Ethical Committee of Gujranwala Medical College, Gujranwala (Approval No.: ME.IRB.697/GMC; dated: 07 June 2024) and the Institutional Ethical Committee of Bacha Khan Medical College, Mardan (Approval No.: 973/ORT/BKMC; dated: 13 June 2024). All participants provided written informed consent. The study was conducted in accordance with the principles of the Declaration of Helsinki, and patient anonymity was strictly maintained.

## Results

The study enrolled a total of 180 patients who presented to the emergency department with non-traumatic acute abdominal pain. As shown in Table 1, the mean age of the study population was 39.5 ± 13.6 years, with participants ranging from 18 to 80 years, reflecting a broad adult age group. Gender distribution was relatively balanced, with a slightly higher number of male patients (94, 52.2%) compared to female patients (86, 47.8%). These baseline characteristics highlight the demographic diversity of the sample, enhancing the generalizability of the results to a typical emergency department setting. The inclusion of both younger and older adults provides a comprehensive overview of POCUS utility across various age groups.

**Table 1:** Descriptive statistics of the study population.

Variable	Value
Number of patients	180
Mean age (±SD), years	39.5 ± 13.6
Age range (years)	18-80
Gender – male	94 (52.2%)
Gender – female	86 (47.8%)

A wide range of abdominal pathologies were represented among the final diagnoses, highlighting the diagnostic challenges associated with acute abdominal pain. As detailed in Table 2, the most common diagnosis was appendicitis, found in 44 (24.4%) patients. Renal colic accounted for 34 (18.9%) cases, followed by cholecystitis in 30 (16.7%) patients. In 28 (15.6%) cases, bowel obstruction was diagnosed. The remaining 44 (24.4%) patients were categorized as “Other,” which included gastroenteritis, nonspecific abdominal pain, or conditions not amenable to ultrasound evaluation. Importantly, patients classified as nonspecific abdominal pain were followed up through imaging, clinical course, or structured telephone review to confirm symptom resolution and to ensure that no significant diagnoses were missed. This diagnostic diversity underscores the need for ultrafast and precise evaluation methods such as POCUS in the emergency setting.

**Table 2:** Distribution of final diagnoses. Final diagnoses were confirmed by a combination of radiology-performed ultrasound, contrast-enhanced CT, operative findings, histopathology, and clinical course within the index visit or on structured follow-up. The “Other” category included heterogeneous conditions, such as nonspecific abdominal pain, gastroenteritis, and miscellaneous causes less amenable to ultrasound diagnosis.

Final diagnosis	Frequency (n)	Percentage (%)
Appendicitis	44	24.4%
Cholecystitis	30	16.7%
Bowel obstruction	28	15.6%
Renal colic	34	18.9%
Other	44	24.4%
Total	180	100%

**Figure 1:**
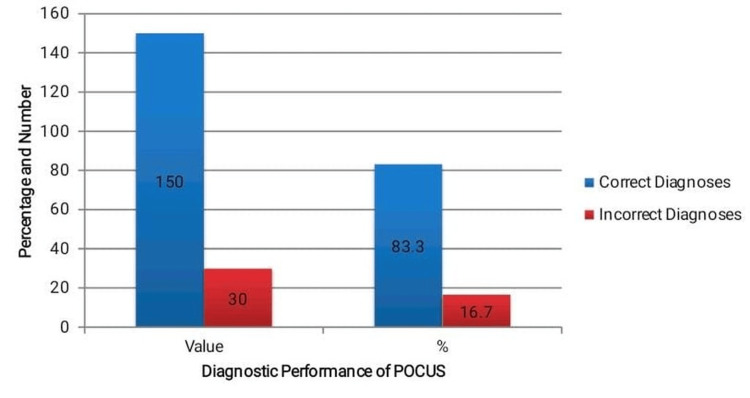
Overall diagnostic performance of point-of-care ultrasound (POCUS).

When the final confirmed diagnoses were used to classify the POCUS performance, a good diagnostic performance of the ultrasound was represented. The overall diagnostic accuracy using POCUS to detect the underlying pathology was high at 83.3% (150 patients out of the 180 were correctly identified), as shown in Figure 1. This highlights the usefulness of POCUS as a first-line imaging modality in emergency rooms, particularly in low-resource environments, where fast decision-making is paramount. The other 30 (16.7) missed diagnoses or false-positive results showed the area of further training or additional diagnosis study.

Since the “Other” category in Table 2 comprised heterogeneous conditions with limited relevance to ultrasound-based evaluation, the subsequent analysis concentrated on the four most frequent and clinically significant diagnoses, i.e., appendicitis, cholecystitis, bowel obstruction, and renal colic, to more accurately assess the diagnostic utility of POCUS. As shown in Table 3, POCUS correctly identified appendicitis in 38 (86.4%) patients, with six (13.6%) false negatives and nine (19.1%) false positives, resulting in an overall accuracy of 92.2%. For cholecystitis, 24 (80.0%) true positives were observed, with five (17.2%) false positives and six (20.0%) false negatives, yielding an accuracy of 93.7%. In bowel obstruction, 22 (78.6%) cases were correctly diagnosed, with only four (15.4%) false positives and six (21.4%) false negatives, showing an accuracy of 94.2%. Renal colic had 29 (85.3%) true positives, six (17.1%) false positives, and five (14.7%) false negatives, achieving an accuracy of 93.9%. These results, with consistently high sensitivity, specificity, and predictive values, underscore the effectiveness of POCUS in accurately diagnosing a wide range of abdominal emergencies.

**Table 3:** Detailed diagnostic metrics by condition. TP: true positive; FP: false positive; TN: true negative; FN: false negative; PPV: positive predictive value; NPV: negative predictive value.

Condition	TP	FP	TN	FN	Sensitivity	Specificity	PPV	NPV	Accuracy
Appendicitis	38	9	123	6	86.4%	93.2%	80.9%	95.3%	92.2%
Cholecystitis	24	5	140	6	80.0%	96.6%	82.8%	95.9%	93.7%
Bowel obstruction	22	4	143	6	78.6%	97.3%	84.6%	96.0%	94.2%
Renal colic	29	6	136	5	85.3%	95.8%	82.9%	96.5%	93.9%

**Figure 2:**
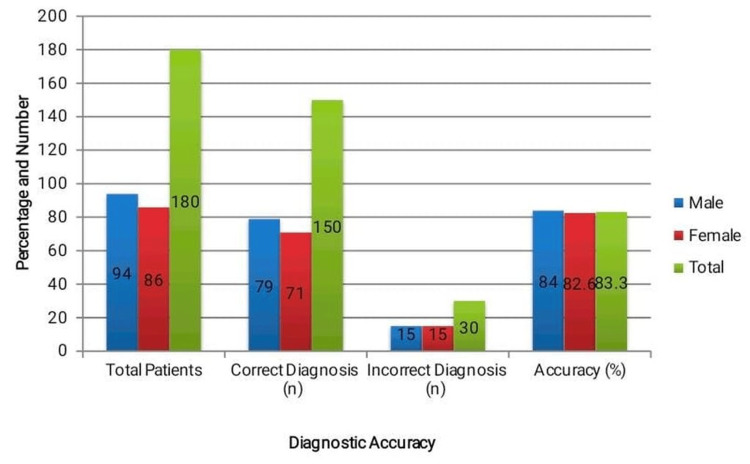
Gender-based point-of-care ultrasound (POCUS) diagnostic accuracy. Male (79/94; 84.0%) vs. female (71/86; 82.6%). The difference is not statistically significant (χ² test, p = 0.79).

In addition to the overall and condition-specific assessments, a gender-based comparison of POCUS diagnostic performance was conducted. Figure 2 shows the diagnostic accuracy as 79 (84.0%) in males and 71 (82.6%) in females. The two sexes had an equal number of incorrect diagnoses: 15 (16.0%) in men and 15 (17.4%) in women. The results state that the difference in the performance of investigation between the genders is not significant, and POCUS is a rather stable and dependable form of investigation regardless of the patient populations.

## Discussion

This research demonstrates that POCUS is a valuable diagnostic modality for assessing acute abdominal pain in the emergency department, with an overall diagnostic accuracy of 83.3%. Sensitivity and specificity values were high for the most common and clinically significant conditions in our cohort, i.e., appendicitis (86.4% and 93.2%, respectively), cholecystitis (80.0% and 96.6%), bowel obstruction (78.6% and 97.3%), and renal colic (85.3% and 95.8%). These findings highlight that POCUS can reliably detect major abdominal emergencies where early recognition is critical. The tool also proved consistent across genders and demonstrated practical feasibility in a resource-constrained emergency setting.

Importantly, while our detailed accuracy analysis focused on these four major diagnoses, POCUS was also applied in patients presenting with a broader range of abdominal pain etiologies. In the “Other” category, comprising heterogeneous conditions such as gastroenteritis, nonspecific abdominal pain, or cases without a clearly identifiable cause, POCUS often served as a useful first-line modality to exclude urgent surgical pathology and support safe clinical decision-making. Although subgroup analyses were not performed for these less common or diagnostically limited conditions due to sample size constraints, their inclusion reflects real-world emergency department practice where POCUS is frequently employed as a rapid triage and screening tool.

When compared to international literature, our findings are consistent with prior studies reporting high diagnostic performance of POCUS in appendicitis, gallbladder disease, and hydronephrosis [19,20]. The high sensitivity and negative predictive value in our study reinforce its role as a valuable screening modality, particularly in time-sensitive clinical scenarios. The observed specificity and positive predictive values indicate that false positives are relatively rare, which enhances clinician confidence in POCUS-guided decision-making. Globally, multiple studies have validated POCUS as a rapid and accurate alternative to formal radiology in emergency settings [21,22], with similar diagnostic performance demonstrated across both high-income and resource-limited healthcare systems [23]. While some reports note lower sensitivity for certain conditions, these differences may reflect operator experience, equipment, or patient demographics [24]. In our study, physicians had a minimum of two years of ultrasound training, which likely contributed to diagnostic consistency.

Unlike most prior research that focuses on single conditions such as gallstones or bowel obstruction, our study provides a broader assessment of POCUS utility across a spectrum of abdominal emergencies. Furthermore, the inclusion of data from a Pakistani emergency department contributes much-needed local evidence to the global literature and helps bridge the gap in data from resource-constrained environments [25].

Limitations and future suggestions

This study has several limitations. First, because it was conducted in only two tertiary care hospitals, the results may not be fully generalizable to other facilities with different patient populations or levels of expertise. Second, convenience sampling introduces the potential for selection bias. Third, although all POCUS scans were performed by trained physicians, inter-observer variability in interpretation may still exist despite quality control measures. Fourth, the “Other” category of abdominal pain was not analyzed in detail; this group included heterogeneous conditions such as gastroenteritis, nonspecific abdominal pain, and cases without a clearly identifiable etiology. In some of these patients, CT imaging was not performed due to clinical improvement or resource limitations, raising the possibility that subtle or less common pathologies were underdiagnosed. Finally, the lack of blinding to clinical presentation may have introduced confirmation bias during image acquisition or interpretation.

Future studies should validate these findings across diverse emergency settings, including rural or resource-limited hospitals. Comparative studies stratified by operator training level may clarify minimum competency requirements and guide curriculum development. Longitudinal research could further define the impact of routine POCUS use on patient outcomes, including time to diagnosis, intervention rates, and cost-effectiveness. Finally, standardized training protocols and objective competency assessments are needed to improve diagnostic reliability and support the broader adoption of POCUS in emergency medicine.

## Conclusions

In this study, POCUS demonstrated high sensitivity, specificity, and overall diagnostic accuracy for selected abdominal emergencies, particularly appendicitis, cholecystitis, bowel obstruction, and renal colic. These findings suggest that POCUS can serve as a valuable adjunct to clinical evaluation in the emergency department, especially in resource-limited settings where rapid decision-making is critical. However, the evidence remains limited to the four conditions assessed, and the possibility of missed or subtle diagnoses cannot be excluded in the absence of long-term follow-up or confirmatory imaging. Therefore, while POCUS shows promise as a supportive diagnostic modality, further multicenter studies with broader diagnostic scope and longitudinal follow-up are needed before its effectiveness in managing undifferentiated abdominal pain can be fully established.
